# Broadening our understanding of genetic risk for scleroderma/systemic sclerosis by querying the chromatin architecture surrounding the risk haplotypes

**DOI:** 10.1186/s12920-021-00964-5

**Published:** 2021-04-24

**Authors:** Kerry E. Poppenberg, Vincent M. Tutino, Evan Tarbell, James N. Jarvis

**Affiliations:** 1 Canon Stroke and Vascular Research Center, Jacobs School of Medicine and Biomedical Sciences, University at Buffalo, Buffalo, NY USA; 2 Department of Neurosurgery, Jacobs School of Medicine and Biomedical Sciences, University at Buffalo, Buffalo, NY USA; 3 Department of Pathology and Anatomical Sciences, Jacobs School of Medicine and Biomedical Sciences, University at Buffalo, Buffalo, NY USA; 4 Department of Biomedical Engineering, University at Buffalo, Buffalo, NY USA; 5 Quantitative Systems Pharmacology, Enhanced Pharmacodynamics, LLC, Buffalo, NY USA; 6 Genetics, Genomics, and Bioinformatics Program, Jacobs School of Medicine and Biomedical Sciences, University at Buffalo, Buffalo, NY USA; 7 Department of Pediatrics, Jacobs School of Medicine and Biomedical Sciences, University at Buffalo, Buffalo, NY USA

**Keywords:** Scleroderma, Systemic sclerosis, Genetic risk, Histone mark, Topologically associated domain

## Abstract

**Background:**

Genetic variants in the human leukocyte antigen (HLA) locus contribute to the risk for developing scleroderma/systemic sclerosis (SSc). However, there are other replicated loci that also contribute to genetic risk for SSc, and it is unknown whether genetic risk in these non-HLA loci acts primarily on the vasculature, immune system, fibroblasts, or other relevant cell types. We used the Cistrome database to investigate the epigenetic landscapes surrounding 11 replicated SSc associated loci to determine whether SNPs in these loci may affect regulatory elements and whether they are likely to impact a specific cell type.

**Methods:**

We mapped 11 replicated SNPs to haplotypes and sought to determine whether there was significant enrichment for H3K27ac and H3K4me1 marks, epigenetic signatures of enhancer function, on these haplotypes. We queried pathologically relevant cell types: B cells, endothelial cells, fibroblasts, monocytes, and T cells. We then identified the topologically associated domains (TADs) that encompass the SSc risk haplotypes in primary T cells to identify the full range of genes that may be influenced by SSc causal SNPs. We used gene ontology analyses of the genes within the TADs to gain insight into immunologic functions that might be affected by SSc causal SNPs.

**Results:**

The SSc-associated haplotypes were enriched (*p* value < 0.01) for H3K4me1/H3K27ac marks in monocytes. Enrichment of one of the two histone marks was found in B cells, fibroblasts, and T cells. No enrichment was identified in endothelial cells. Ontological analyses of genes within the TADs encompassing the risk haplotypes showed enrichment for regulation of transcription, protein binding, activation of T lymphocytes, and proliferation of immune cells.

**Conclusions:**

The 11 non-HLA SSc risk haplotypes queried are highly enriched for H3K4me1/H3K27ac-marked regulatory elements in a broad range of immune cells and fibroblasts. Furthermore, in immune cells, the risk haplotypes belong to larger chromatin structures encompassing genes that regulate a wide array of immune processes associated with SSc pathogenesis. Though importance of the vasculature in the pathobiology of SSc is widely accepted, we were unable to find evidence for genetic influences on endothelial cell function in these regions.

**Supplementary Information:**

The online version contains supplementary material available at 10.1186/s12920-021-00964-5.

## Background

Scleroderma, or systemic sclerosis (SSc), is a spectrum of human diseases characterized by sclerosis involving multiple organs, most prominently the skin and gastrointestinal tract [1], and is accompanied by distinct patterns of autoantibody production [2]. SSc is frequently accompanied by clinically-prominent vascular manifestations such as Raynaud’s phenomenon [3, 4], as well as prominent signs of microvascular injury to a broad range of tissues including the heart and lungs [5]. These clinical manifestations, in addition to multiple lines of clinical and basic research, suggest a complex pathogenesis involving interactions between the immune system, the microvasculature, and fibroblasts [6]. The question remains as to which of these three different features is the primary driver of the disease. Answering this question is of considerable importance, as it would facilitate the search for new and effective therapies.

One way of gaining insight into whether the immune, fibrotic, or vascular phenomena are primary in SSc pathogenesis is to determine where genetic effects that confer risk for the disease are exerted, i.e., vasculature, the immune system, or fibroblasts. Genetic variants have long thought to contribute to the risk for SSc. For example, associations between the human leukocyte antigen (HLA) locus and SSc are well recognized [7]. Furthermore, while SSc disease prevalence is quite low (50–300 cases per million or no more than 0.03% of the population), the disease prevalence in siblings of affected individuals may be as high as 2.6% [8]. Several genome-wide association studies (GWAS) have been performed on patients with SSc, and, predictably, the HLA locus has been replicated as the most important region for genetic risk in all of these studies [9–12]. While these data provide evidence that the immune system may be a primary driver of the clinical entity we call SSc, there are at least 11 other loci outside of the HLA locus that have been replicated in at least 2 independent studies [8]. We therefore asked whether there was any evidence that genetic effects at these loci might be exerted on vascular, rather than immune cells.

To accomplish this task, we used an approach similar to that taken in our previously-reported investigations of juvenile idiopathic arthritis [13], systemic lupus [14], and intracranial aneurysm [15]. We queried the 11 replicated, non-HLA loci associated with SSc to determine whether these loci were enriched for regulatory elements that are generally thought to be involved in autoimmune disease risk [16, 17], mining publicly available data on a variety of related cell types. Furthermore, we examined the broader chromatin architecture surrounding the SSc risk haplotypes to gain further insight into the mechanisms through which variants in these 11 haplotypes might confer disease risk.

## Methods

### Defining LD blocks

We queried 11 single nucleotide polymorphisms (SNPs) in non-HLA loci with established and replicated associations with SSc identified by GWAS and genetic fine mapping studies and reported in Korman et al. [8]. We then used the SNiPA online single nucleotide polymorphism annotator [18] (https://snipa.helmholtz-muenchen.de/snipa3/) to define linkage disequilibrium (LD) blocks for each of the 11 SNPs. We used the following settings: GRCh37, 1000 Genomes Phase 3 v5, querying European populations, and setting r^2^ at 0.8. The smallest genomic position was used as the start of the LD block while the largest position was used as the end of the block. The liftover tool from UCSC was used to convert the positions to GRCh38. These regions are presented in Table 1.

**Table 1:** Positional information of the 11 scleroderma-risk single nucleotide polymorphisms and the associated haplotypes

SNP	Haplotype	SNP location	Locus name
rs2205960	chr1:173222336–173287411	Intergenic	*TNFSF4*
rs10488631	chr7:128945562–129071820	Intergenic	*IRF5*
rs11642873	chr16: 85956661–85958099	Intergenic	*IRF8*
rs2056626	chr1:167448647–167467063	Intronic	*CD247*
rs3790567	chr1:67340123–67361333	Intronic	*IL12RB2*
rs3821236	chr2:191035723–191071078	Intronic	*STAT4*
rs77583790	chr3:159907604–159976265	Intronic	*SCHIP1-IL12A*
rs9373839	chr6:106181815–106339294	Intronic	*ATG5*
rs5029939	chr6:137849452–137921300	Intronic	*TNFAIP3*
rs1378942	chr15:74751897–74821981	Intronic	*CSK*
rs2305743	chr19:18068862–18092777	Intronic	*IL12RB1*

SNPs from Korman et al. [8] were mapped to haplotype blocks using SNiPA. SNP location was determined using the UCSC Genome Browser

*chr* chromosome, *rs* reference SNP, *SNP* single nucleotide polymorphism

### Identification of H3K4me1/H3K4me3/H3K27ac histone marks within LD blocks

In the 11 risk haplotypes, we assessed the presence of H3K4me1 and H3K27ac histone marks, which typically identify weak and strong enhancers respectively, within the 11 risk haplotypes. We also examined H3K4me3 marks to identify promoters. We queried H3K4me1/H3K4me3/H3K27ac ChIPseq data available from the Cistrome database (http://cistrome.org/db/#/) from multiple cell types: CD19+ B lymphocytes, fibroblasts, human umbilical vein endothelial cells (HUVECs), CD14+ monocytes, and CD4+ T lymphocytes. The Cistrome dataset information is provided in Table 2.

**Table 2:** Cistrome dataset information

Publication	Cell type	Cell population	Factor	GEO accession
Kim DE, 2017	Fibroblast	Primary dermal fibroblasts	H3K27ac	GSM2151910
Adoue V, 2014	Fibroblast	Fibroblast primary cell line	H3K4me1	GSM1435528
Adoue V, 2014	Fibroblast	Fibroblast primary cell line	H3K4me3	GSM1435529
Andersson R, Nature 2014	B Lymphocyte	CD19+	H3K27ac	GSM998997
Andersson R, Nature 2014	B Lymphocyte	CD19+	H3K4me1	GSM998994
Bernstein BE, Nat. Biotechnol 2010	B Lymphocyte	CD19+	H3K4me3	GSM1027300
Andersson R, Nature 2014	T Lymphocyte	CD4+ (strain naïve)	H3K27ac	GSM999004
Andersson R, Nature 2014	T Lymphocyte	CD4+ (strain naïve)	H3K4me1	GSM999003
LaMere SA, Genes Immun 2016	T Lymphocyte	CD4+ (strain naïve)	H3K4me3	GSM1888744
ENCODE Project Consortium, Nature 2012	Endothelial Cell	HUVEC	H3K27ac	GSM733691
ENCODE Project Consortium, Nature 2012	Endothelial Cell	HUVEC	H3K4me1	GSM733690
ENCODE Project Consortium, Nature 2012	Endothelial Cell	HUVEC	H3K4me3	GSM733673
ENCODE Project Consortium, Nature 2012	Monocyte	CD14+	H3K27ac	GSM1003559
ENCODE Project Consortium, Nature 2012	Monocyte	CD14+	H3K4me1	GSM1003535
ENCODE Project Consortium, Nature 2012	Monocyte	CD14+	H3K4me3	GSM1003536

We used the intersect command within the BEDTools software package to identify intersections between H3K4me1/H3K4me3/H3K27ac peak data and linkage disequilibrium LD regions (haplotypes) for each cell type, as described by Jiang et al. [19]. To determine significance, we created 11 random regions with length equal to the average length of the 11 LD blocks of interest (59,976 bp). Using bedtools intersect, we determined how many random regions overlapped with the peak file for a given cell type and H3K4me1/H3K4me3/H3K27ac enrichment. We repeated this process 1000 times to approximate a normal distribution. We then determined where the number of overlaps with the regions of interest fell within the normal curve in order to calculate the associated *p* value. Enrichment for histone post-translational modifications (H3K4me1/H3K4me3/H3K27ac) showing a *p* value < 0.01 was considered statistically significant.

We also examined expression of locus genes for cell types with significant enrichment for both enhancer marks using primary cell RNA sequencing data. Genes were considered expressed if they had TPM > 1 in at least half of samples analyzed.

### Identification of relevant topologically associated domains in CD4+ T cells

**Fig. 1:**
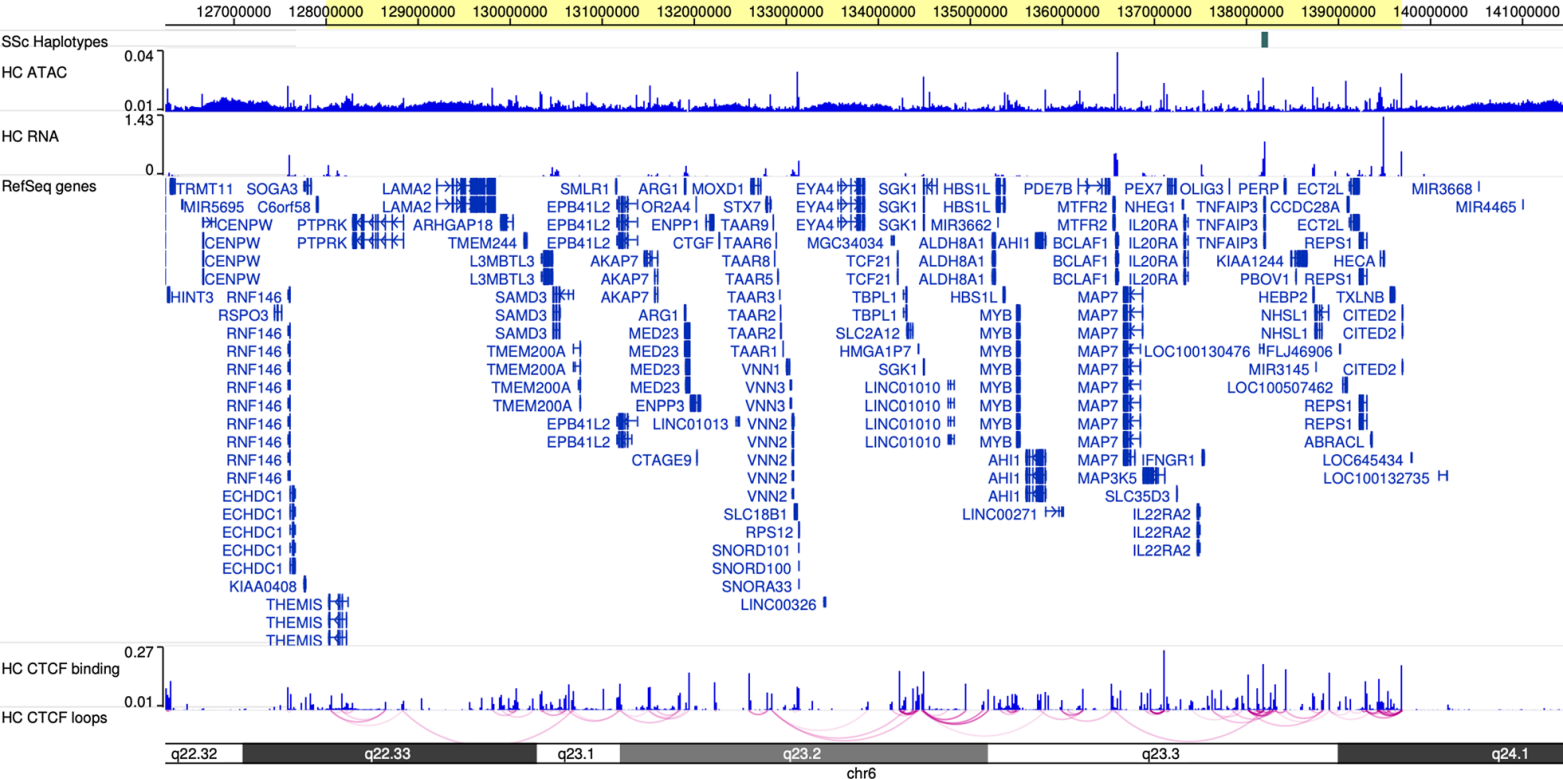
WashU genome browser visualization of chromatin loops within primary CD4+ T cells surrounding TNFAIP3 locus. Yellow highlight at top indicates merged loops that form TAD. Next track shows the haplotype encompassed by the TAD, followed by ATACseq and RNAseq data from healthy CD4+ T cells. Refseq track shows all genes within the region. Last tracks illustrate HiChIP loops for healthy controls

We next sought to identify potential gene targets of H3K4me1/H3K27ac-marked enhancers on the SSc-associated risk haplotypes. While enhancers may not regulate the nearest gene, they typically regulate genes within the same chromatin loop or topologically associated domain (TAD) [20–22]. We used previously published CTCF-HiChIP data from primary CD4+ T cells to identify CTCF loop anchors within the SSc risk haplotypes [23]. Although these data were generated in pediatric samples, they provide detailed view of 3D chromatin structures in a relevant primary human cell. HiChIP loops were converted into paired end BED files; only loops supported by two or more reads were retained. If an LD block did not contain a CTCF loop anchor, it was considered inactive in CD4+ T cells and excluded. If an LD block did contain a CTCF loop anchor as identified on ChIPseq performed on the same samples, we determined all genomic regions that physically interacted with the block. We then identified any regions that interacted with those anchors, and continued this process until all regions demonstrating close physical proximity with the LD block were identified. Further, we used bedtools merge to merge all anchors with a maximum gap between anchors of 1 MB to define positions for the associated TADs. Figure 1 illustrates an example of CTCF loops and binding surrounding TNFAIP3 locus. Next, we used the UCSC genome browser to identify genes within the TADs of the 11 replicated SSc-associated risk haplotypes. We determined whether these genes were expressed in healthy control CD4+ cells at level of TPM > 1 in at least 50% of samples. Only genes found to be expressed in CD4+ were used for ontological analyses.

**Fig. 2:**
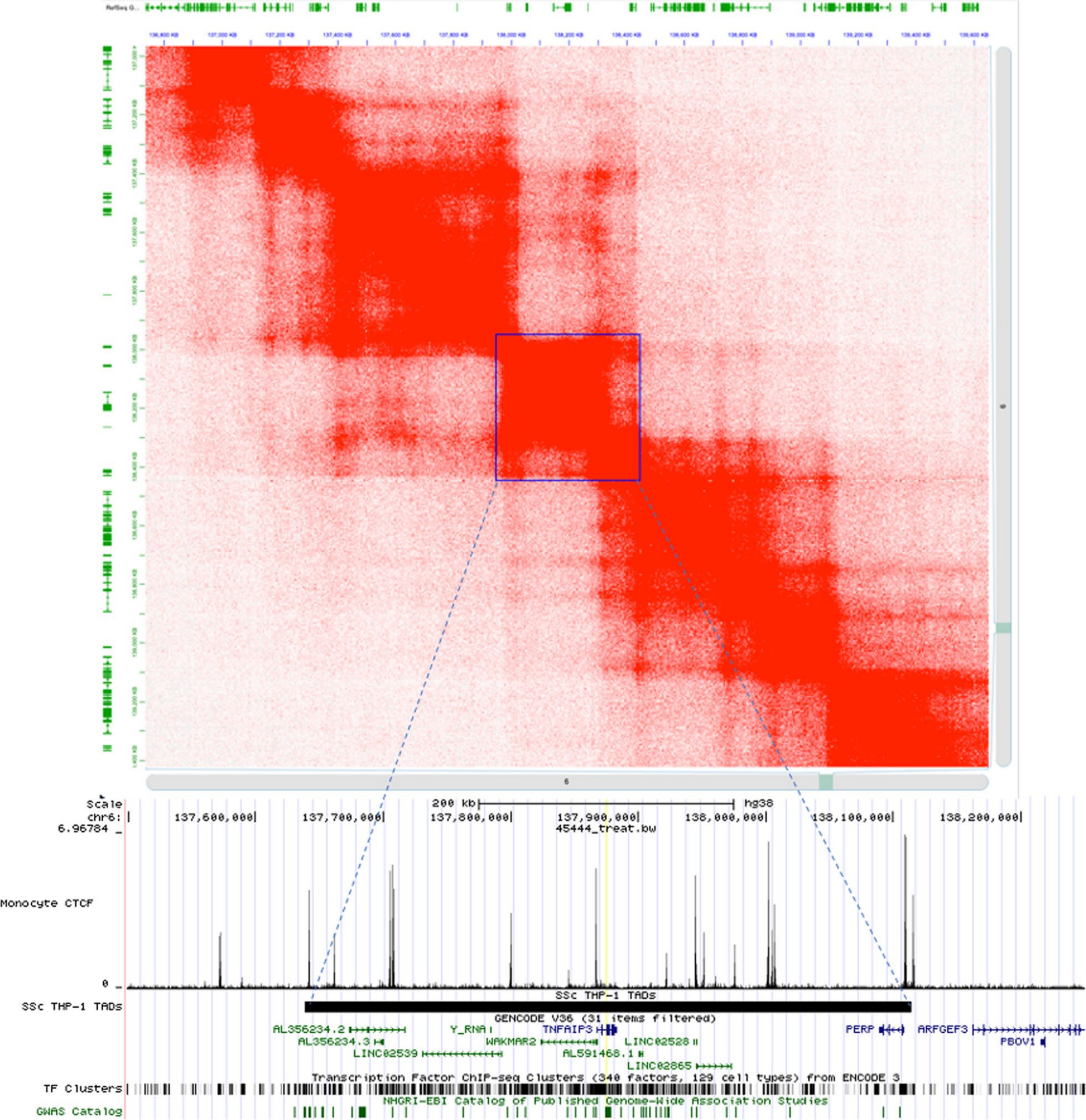
Juicebox defined TAD for the TNAIP3 locus with CTCF ChIP-seq data in monocytes to corroborate TAD boundaries. HiC map of THP-1 cells used to identify TAD (blue box). Green lines at top and left represent RefSeq genes. TAD coordinates compared to monocyte CTCF peaks to verify if TAD defined correctly

Due to lack of primary cell data, we took a different approach to identify TADs in monocytes. We used the publicly available Juicebox software [24] to identify TADs in THP-1 monocytes using data from Phanstiel et al. [25]. We applied the balanced normalization to each Hi-C map to correct for any experimental bias, per author’s recommendation [24]. We used a 5 KB resolution to visualize chromatin loops when identifying SSc TADs. We loaded RefSeq genes via the 1D annotation panel to determine which genes were encompassed within the TAD. We used the straight edge tool to precisely locate the haplotype and then to identify the exact chromosomal positions of the TAD as displayed in the information pane. As TADs are defined visually, which may differ from user to user, we verified our analyses by querying monocyte CTCF ChIP-seq data downloaded from Cistrome under accession number GSM1003508 (Liftover tool was used to convert TAD coordinates from Juicebox from hg19 to hg38 to match CTCF data) to assure that the identified TADs had the predicted CTCF anchors on each side. Figure 2 shows an example of the landscape around TNFAIP3; the upper portion depicts the TAD defined in Juicebox and the lower browser window shows the CTCF peaks along with the TAD boundaries. The TADs with CTCF peaks at the boundaries were considered to be true TADs, and the genes encompasses within the TAD were recorded. CTCF peaks within the TAD structure represent subloops. As done with T cells, genes with expression level of TPM > 1 in at least 50% of healthy monocyte samples were used for ontological analyses.

### Identification of ontologies and functions of genes within TADs

We performed gene ontology enrichment analysis on expressed genes within TADs in CD4+ T cells compared against a background of all protein coding genes, obtained from biomart ensembl, using the public Gene Ontology enRIchment anaLysis and visuaLizAtion tool (GORILLA) [26]. We further used Reduce Visualize Gene Ontology (REVIGO) tool to reduce ontologies based on semantic similarity measures [27]. To better understand potential mechanisms associated with these potential target genes, we used Ingenuity Pathway Analysis (IPA) to identify top disease and biological functions [28]. Functions with a Benjamini–Hochberg *p* value < 0.01 that corresponded to 5 or more input genes were reported.

## Results

### H3K27ac and H3K4me1 enrichment within the SSc LD blocks

**Fig. 3:**
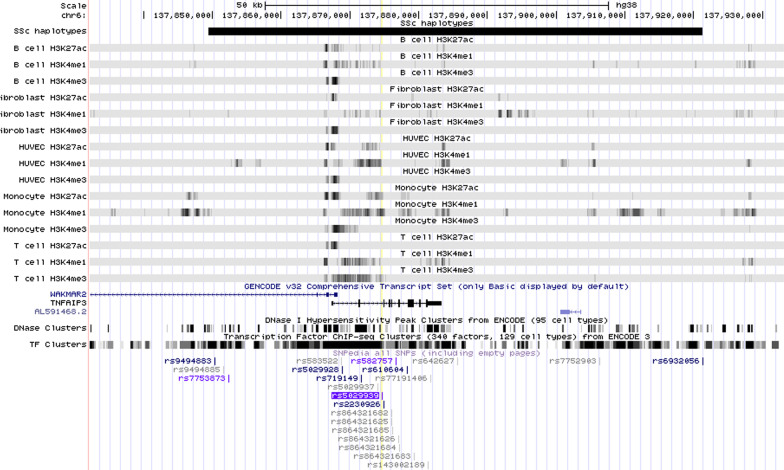
UCSC Genome browser visualization of landscape surrounding TNFAIP3 locus. The yellow vertical line indicates position of the index SNP (rs5029939). Black horizontal bar at the top represents the haplotype blocks of the associated SSc-risk SNPs. The subsequent tracks as progress down are the bigWig files provided by Cistrome (hg38) for H3K27ac, H3K4me1, and H3K4me3 marks in B cells, fibroblasts, HUVECs, monocytes, and T cells. Gene annotation set from GENCODE v32 is presented below histone tracks. Beneath that, the two rows of black vertical lines depict DNase hypersensitivity clusters in 95 cell types from ENCODE and transcription factor ChIP clusters of 340 factors from ENCODE. SNPedia SNPs are presented at the bottom

We used Cistrome H3K27ac and H3K4me1 ChIPseq data to query the SSc-associated LD blocks to determine whether the disease-associated haplotypes are enriched (compared to randomly-selected regions of the genome) for these functional marks in pathologically relevant cell populations. We also queried presence of H3K4me3 marks. Table 3 reports the presence or absence of H3K27ac, H3K4me1, and H3K4me3 marks in the 11 LD blocks of interest for all cell types. These marks can be visualized as individual tracks for the cells queried in the UCSC Genome Browser. For example, Fig. 3 depicts the chromatin landscape surrounding rs5029939 in which we can see histone marks present within the haplotype. Landscape figures for other loci are provided in Additional file 1.

**Table 3:** Histone marks present in scleroderma-associated haplotypes

Haplotype Block	SNP	B cell			Fibroblast			HUVEC			Monocyte			T cell		
		H3K27ac^†^	H3K4me1	H3K4me3	H3K27ac	H3K4me1^†^	H3K4me3	H3K27ac	H3K4me1	H3K4me3	H3K27ac^†^	H3K4me1^†^	H3K4me3^†^	H3K27ac^†^	H3K4me1	H3K4me3
*p* val		0.002	0.018	0.186	0.030	0.006	0.015	0.041	0.068	0.180	0.003	0.007	0.010	0.008	0.025	0.089
chr1:67340123–67361333	rs3790567	–	–	–	–	Y	–	–	–	–	–	–	–	–	–	–
chr1:167448647–167467063	rs2056626	–	Y	Y	Y	Y	Y	–	Y	Y	Y	Y	Y	Y	Y	–
chr1:173222336–173287411	rs2205960	–	–	–	Y	Y	–	Y	Y	–	–	Y	–	–	–	–
chr2:191035723–191071078	rs3821236	–	–	–	Y	Y	–	Y	Y	–	Y	Y	Y	–	Y	–
chr3:159907604–159976265	rs77583790	Y	Y	Y	Y	Y	–	Y	Y	–	Y	Y	Y	–	–	Y
chr6:106181815–106339294	rs9373839	Y	Y	Y	Y	Y	Y	Y	Y	Y	Y	Y	Y	Y	Y	–
chr6:137849452–137921300	rs5029939	Y	Y	Y	Y	Y	Y	Y	Y	Y	Y	Y	Y	Y	Y	–
chr7:128945562–129071820	rs10488631	Y	Y	Y	Y	Y	Y	Y	Y	Y	Y	Y	Y	Y	Y	Y
chr15:74751897–74821981	rs1378942	Y	Y	Y	Y	Y	Y	Y	Y	Y	Y	Y	Y	Y	Y	Y
chr16:85956661–85958099	rs11642873	–	–	–	–	–	–	–	–	–	–	–	–	–	–	–
chr19:18068862–18092777	rs2305743	Y	Y	Y	Y	Y	–	–	–	–	Y	Y	Y	Y	Y	Y

“Y” indicates histone mark for given cell type was present within that haplotype block; “–” if mark is absent. Marks that occur at statistically greater than expected frequency (*p* < 0.01) are denoted with ^†^

*chr* chromosome, *rs* reference SNP cluster ID, *SNP* single nucleotide polymorphism, *Y* yes, *HUVEC* human umbilical vein endothelial cell

While both H3K4me1/H3K27ac marks were found in the majority of LD blocks for all cell types, only monocytes showed significant enrichment by empirical p-value calculation for both epigenetic marks (*p* = 0.003 for H3K27ac, *p* = 0.004 for H3K4me1) compared to the background. It is important to note that H3K4me3 marks were also significantly enriched within the haplotypes in monocytes (*p* = 0.010), and this enrichment may reflect genetic influences on promoters in these cells. We found that 10 of 11 locus genes (excluding SCHIP-IL12A) were expressed in monocytes when querying publicly available RNA sequencing data (GSE147608, n = 11). We also identified significant enrichment for H3K27ac marks (but not H3K4me1) in B cells (*p* = 0.003) and CD4+ T cells (*p* = 0.007), and significant enrichment for H3K4me1 (but not H3K27ac) marks in fibroblasts (*p* = 0.008). Neither mark was enriched in HUVECs.

### Ontological analysis of genes within TADs of CD4+ T cells

H3K4me1/H3K27ac histone marks identify regions that have a strong likelihood of exhibiting enhancer function. However, enhancers do not always regulate the most proximal gene (in terms of linear genomic distance). Gasperini et al. [22] have shown that > 70% of genes regulated by enhancers lie within the same TAD as the enhancers, and that regulatory effects outside the TAD are generally weaker than those within the TAD.

**Fig. 4:**
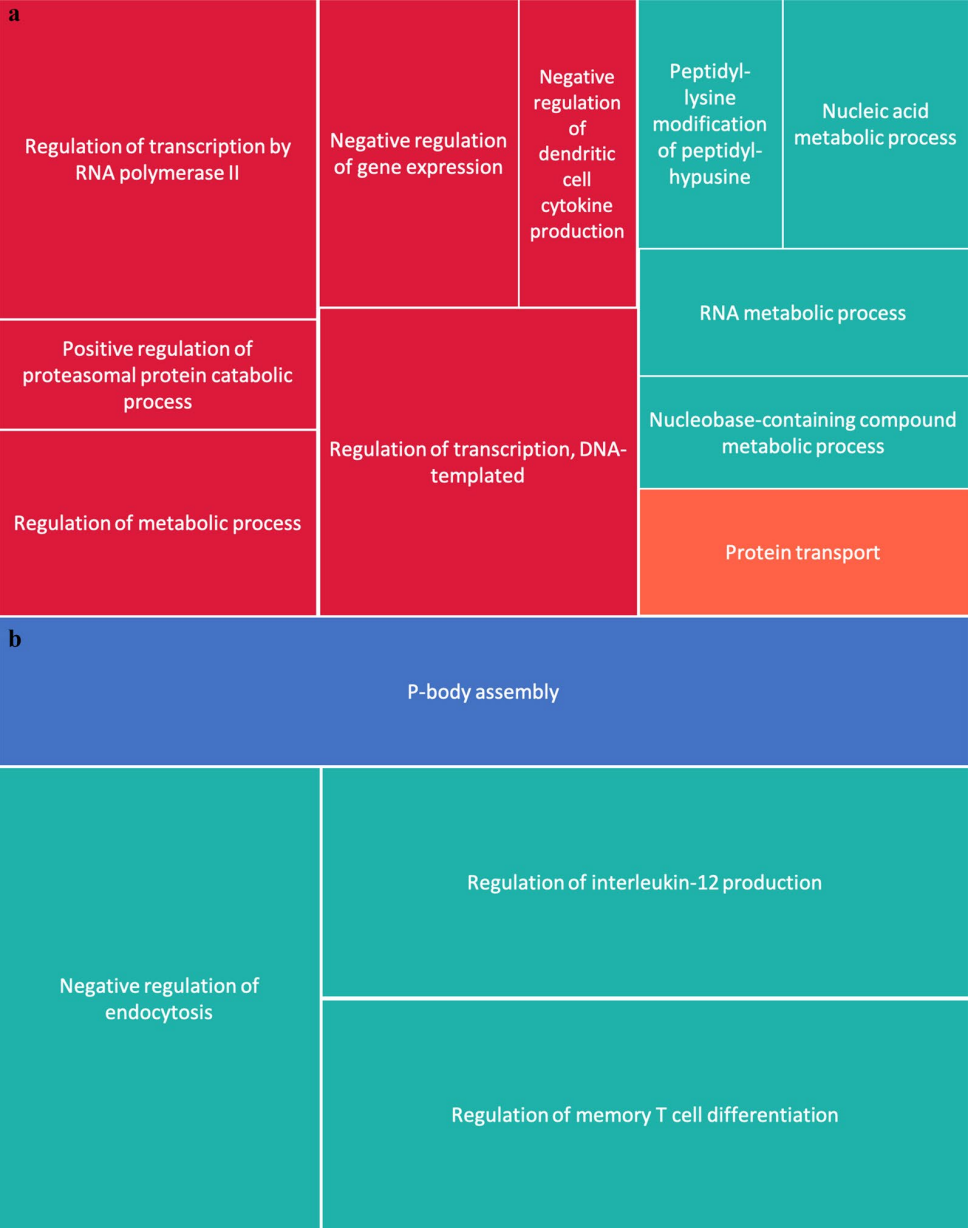
REVIGO treemaps of biological processes associated with expressed genes within CD4+ and monocyte TADs surrounding SSc loci. **a** Reduced biological processes for genes expressed within CD4+ TADs. **b** Reduced biological processed for genes expressed within monocyte TADs

First, we examined T cells using HiChIP-defined TADs from CD4+ primary cell data. CTCF loop anchors were not present in 4 of the SSc-associated haplotypes. For the haplotypes in which CTCF anchors were present, we identified a total of 854 genes. Of those genes, 502 were expressed in CD4+ T cells of healthy individuals. We then identified 41 associated biological processes, including regulation of transcription, protein transport, and regulation of metabolic process. A reduced list of these terms generated using REVIGO is presented as a treemap in Fig. 4a. Next, we surveyed molecular functions and found 21 significant functions associated with the genes expressed within the relevant CD4+ T cells TADs. These functions included transcription regulator activity, DNA binding, protein binding. Additional file 2 presents the full list of GORILLA results. We further used IPA to examine top diseases and biological functions for the refined set of genes that were expressed in CD4+ T cells. We used a Benjamini–Hochberg *p* value < 0.01 and the requirement that at least five of the input genes were assigned to the annotation to identify significant terms. There were 92 annotations reported, including activation of T lymphocytes, proliferation of immune cells, transcription of RNA, and binding of DNA. See Additional file 3 for full significant results from IPA.

Next, we investigated the ontologies associated with genes expressed within monocyte TADs. All 11 TADs defined visually in Juicebox also had CTCF peaks present at those boundaries and encompassed 113 genes, of which 59 genes were expressed in healthy monocytes. These genes associated with 19 biological processes, predominantly focused on cytokine production and signaling. The reduced terms identified by REVIGO are presented in a treemap in Fig. 4b. Only one molecular function, RNA polymerase II core promoter sequence-specific DNA binding, was identified. Full lists for associated biological processes and molecular functions are provided in Additional file 2. Using IPA, we identified 43 significant disease and biological functions, including activation of lymphocytes, systemic lupus erythematosus, and cytotoxicity of cells. The full list is presented in Additional file 3.

## Discussion

It is becoming increasingly clear that, for most complex genetic traits, including autoimmune diseases, genetic risk is largely exerted on regulatory elements that control gene expression rather than on the protein-coding sequences of relevant genes [16, 29]. These regulatory regions, and their most likely target genes, can be identified based on specific features of the surrounding chromatin. Although these features are not *prima facie* evidence that the region of interest has regulatory function [21], they can be useful guides to assess probable genetic mechanisms. Therefore, in this study we used the broader chromatin architecture encompassing the established and replicated SSc haplotypes as a means of investigating the mechanisms through which genetic variants might confer disease risk.

Using ChIPseq data accessed through the Cistrome database, we queried H3K27ac/H3K4me1 enrichment in 11 replicated SSc risk haplotypes in multiple pathologically relevant cell types: B cells, fibroblasts, HUVECs, monocytes, and T cells. We found evidence for genetic influences on regulatory regions in immune cells, particularly monocytes (enriched for both H3K27ac and H3K4me1) and fibroblasts (enriched for H3K4me1, which typically marks poised rather than active enhancers), and CD4+ T cells (enriched for H3K27ac, a marker for active enhancers). However, we found no evidence that genetic risk operates on the vasculature, as neither epigenetic mark was significantly enriched in HUVEC compared with genome background. Furthermore, examining expressed genes found in the TADs that encompass the SSc risk haplotypes using primary CD4+ data, we identified multiple expression related cellular processes (e.g., regulation of gene expression, regulation of transcription by RNA polymerase II, protein transport).

Examining the IPA-annotated disease and biological functions in CD4+ T cells, we found proliferation of immune cells and activation of T lymphocytes were among the significant, further corroborating the pathologic relevance of these analyses. Additional refinement of these data, for example, by performing gene expression experiments in a well-characterized and genotyped population, may be a promising way to identify previously unsuspected targets of therapy for SSc. We also note that cancer is one of the most significant disease categories that emerged from the IPA analysis of the genes in CD4+ T cells TADs. There is an increased risk of malignancy in patients with SSc and some suggest that SSc is related to an immune response against cancer [30]. Gene profiling by Dolcino et al. also reflected an oncogenic gene signature [31]. We note 38 of the genes found expressed in T cells TADs encompassing the scleroderma associated SNPs were also found to be differentially expressed in Dolcino’s study [31], including BSG, FCER2, GPA33, MAP2K7, SCAMP2, and TSPAN33.

It is interesting that neither H3K4me1 nor H3K27ac is enriched in the SSc haplotypes in HUVECs, given the abundant evidence that the microvasculature is the primary target of injury in this disease. One of first clinical signs of SSc is Raynaud’s phenomenon, the loss of normal regulation of cutaneous vessels, which affects thermoregulation. The areas most afflicted by impaired thermoregulation, predominantly the extremities, are also the regions with most progressed skin fibrosis [5]. Raynaud’s phenomenon is thought to result from endothelial injury. Another clinical sign of SSc is abnormal capillary structure, which reflects microvascular damage that may be due to both neovascularization and loss of vascularization due to impaired blood flow [32]. Following microvascular injury, as the disease progresses, there is further involvement of endothelium and blood vessels within multiple organs (heart, lungs, kidneys, and GI tract), resulting in tissue ischemia, fibrosis, and organ failure [5]. Vascular remodeling and subsequent tissue fibrosis are the next stages in pathogenesis, which further suggests that the vasculature is the main target in SSc. However, our data do not allow us to conclude that genetic influences are the primary driver of these well-documented vascular phenomena in SSc.

A novel finding in this study is the implication of monocytes, which were highly enriched for H3K4me1/H3K27ac marks within the SSc risk haplotypes. It is known that monocytes are among the infiltrates following endothelial injury in SSc [33]. Monocytes can produce fibroblasts [34], which are critical in the progressive fibrosis indicative of scleroderma, and can differentiate into macrophages, an important cell in tissue homeostasis and inflammation [35]. Both cell types have reported abnormalities in scleroderma [36, 37]. It has also been suggested that there is a profibrotic phenotype expressed by circulating monocytes in SSc patients [38]. Monocytes have been associated with fibrotic disease; for instance, elevated monocyte count has been reported as a biomarker for poor outcome [39, 40]. We also found H3K4me3 marks to be enriched on the SSc haplotypes in monocytes, which, as we noted previously, may reflect genetic influences on active promoters in these cells. There is a clear need to further study monocytes and how potential genetic risk is conferred through these cells in SSc. More detailed maps of chromatin architecture in monocytes are clearly needed. The emergence of genomic technologies such as Cleavage Under Targets and Release Using Nuclease (CUT&RUN [41]), which allow DNA–protein interactions to be profiled on as few as 100,000 cells, now make the generation of such maps technically feasible.

We note several limitations in this study. The first concerns our assessment of genetic influences on vascular function using data from HUVEC, which are an imperfect model. It is possible that if we were to examine endothelial cells from other tissues (e.g., dermal microvascular endothelial cells), we might obtain other results. Unfortunately, we were unable to find ChIPseq data from these cells on public databases. A second limitation is the fact that we focused our analyses on a limited number of risk loci, i.e., only those that have been rigorously replicated in a European population. A total of 34 regions have been identified in different studies in a broad range of populations [6], and we may therefore be underestimating genetic influences on immune cells, fibroblasts, or the endothelium, or overlooking genetic influences that may be exerted in non-European populations. We expanded our analysis to include these additional regions (33 regions studied as 1 failed to map) and found no significant enrichment in these regions (*p* < 0.01) for the functional marks that we queried. We note, however, that H3K27ac in monocytes, and H3K4me1 and H3K4me3 in HUVECs did achieve *p* values < 0.05. See expanded table in Additional file 4. This difference in results from testing done with the 11 validated regions is expected as the added SNPs have not been confirmed by an independent study and some will likely fail in future validation studies. These findings illustrate the importance of being cautious regarding conclusions about genetic mechanisms based on regions that have not been validated.

Finally, we note that our investigations into TAD structure and the genes in CD4+ T cells within them were generated from pediatric samples. There may be small differences between our findings and what would be seen in adult CD4+ T cells, although recently published data demonstrate considerable conservation of the broader chromatin architecture across immune cells and immune cells lines [42].

## Conclusions

The 11 replicated, non-HLA risk haplotypes for SSc are highly enriched for H3K4me1/H3K27ac-marked regulatory elements in a broad range of immune cells and fibroblasts. Furthermore, in CD4+ T cells, the risk haplotypes are parts of larger chromatin structures that include multiple genes that regulate a broad spectrum of immune processes relevant to SSc pathobiology. We were unable to find evidence for genetic influences on endothelial cell function, although replication of additional putative risk loci may reveal pathologically relevant genetic influences on these cells.

## Supplementary Information

**Additional file 1.** UCSC Genome browser of chromatin landscape surrounding SSc-risk loci of interest. The yellow vertical line indicates position of the index SNP. Black horizontal bar at the top represents the haplotype block of the associated SSc-risk SNPs. The subsequent tracks as progress down are the bigWig files provided by Cistrome (hg38) for H3K27ac, H3K4me1, and H3K4me3 marks in B cells, fibroblasts, HUVECs, monocytes, and T cells. Gene annotation set from GENCODE v32 is presented below histone tracks. Beneath that, the two rows of black vertical lines depict DNase hypersensitivity clusters in 95 cell types from ENCODE and transcription factor ChIP clusters of 340 factors from ENCODE. SNPedia SNPs are presented at the bottom.

**Additional file 2.** Significant biological process, molecular function, and cellular component GORILLA ontologies.

**Additional file 3.** Significant disease and biological function IPA annotations.

**Additional file 4.** Histone marks present in expanded scleroderma-associated haplotypes.

## Data Availability

The datasets analyzed during the current study are available on the Cistrome database [http://cistrome.org/db/#/] and Juicebox [http://aidenlab.org/juicebox/].

## Abbreviations

GORILLA: Gene Ontology enRIchment anaLysis and visuaLizAtion
GWAS: Genome-wide association study
HLA: Human leukocyte antigen
HUVEC: Human umbilical vein endothelial cell
IPA: Ingenuity pathway analysis
LD: Linkage disequilibrium
REVIGO: Reduce visualize gene ontology
SNP: Single nucleotide polymorphism
SSc: Systemic sclerosis
TAD: Topologically associated domain
