# Supplementary material for: Broadening our understanding of genetic risk for scleroderma/systemic sclerosis by querying the chromatin architecture surrounding the risk haplotypes
Source: BMC Med Genomics. 2021 Apr 24;14:114. doi: 10.1186/s12920-021-00964-5 (PMC8066847; doi:10.1186/s12920-021-00964-5)
Supplement: Supplementary file 1 — Additional file 1. UCSC Genome browser of chromatin landscape surrounding SSc-risk loci of interest. The yellow vertical line indicates position of the index SNP. Black horizontal bar at the top represents the haplotype block of the associated SSc-risk SNPs. The subsequent tracks as progress down are the bigWig files provided by Cistrome (hg38) for H3K27ac, H3K4me1, and H3K4me3 marks in B cells, fibroblasts, HUVECs, monocytes, and T cells. Gene annotation set from GENCODE v32 is presented below histone tracks. Beneath that, the two rows of black vertical lines depict DNase hypersensitivity clusters in 95 cell types from ENCODE and transcription factor ChIP clusters of 340 factors from ENCODE. SNPedia SNPs are presented at the bottom. [file 12920_2021_964_MOESM1_ESM.docx]

**Additional File 1. UCSC Genome browser screenshots of landscapes surrounding SSc-risk loci on interest.**

1. **TNFSF4**

**
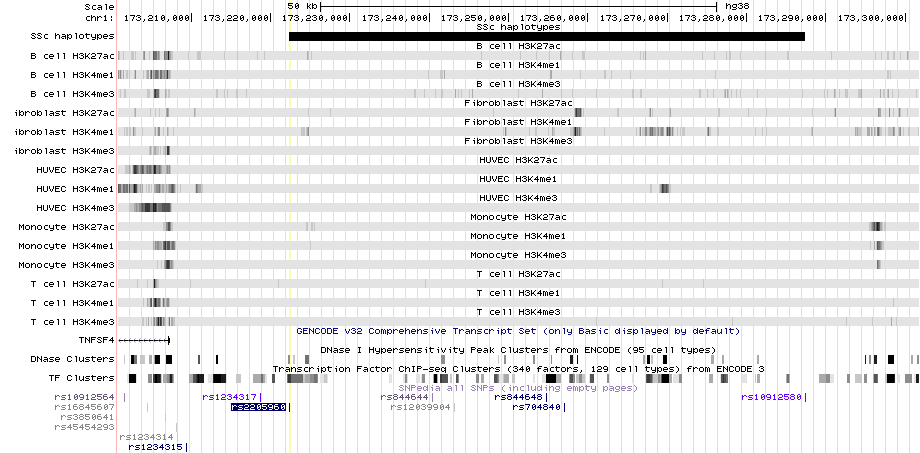
**

1. **IRF8**

**
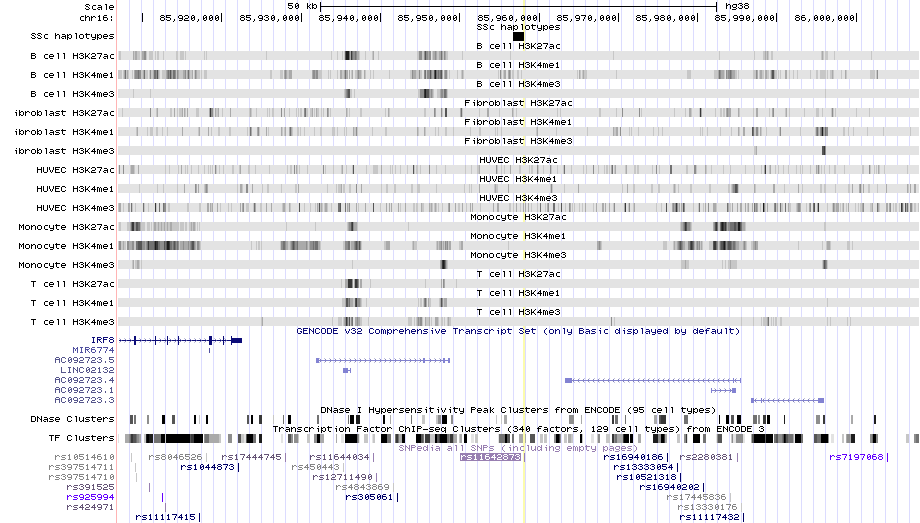
**

1. **CD247**

**
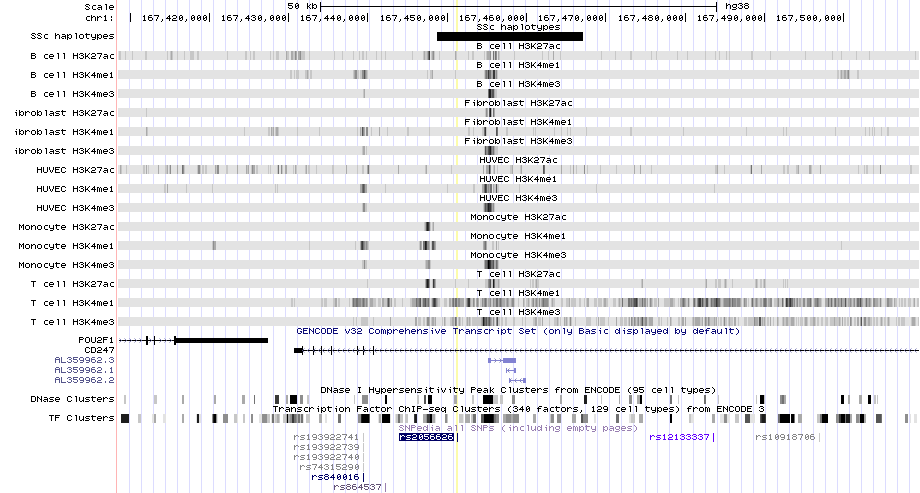
**

1. **IL12RB2**

**
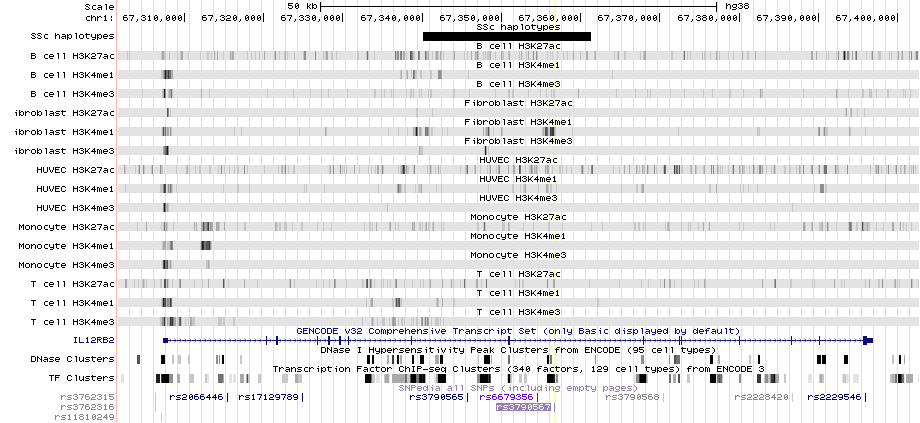
**

1. **STAT4**

**
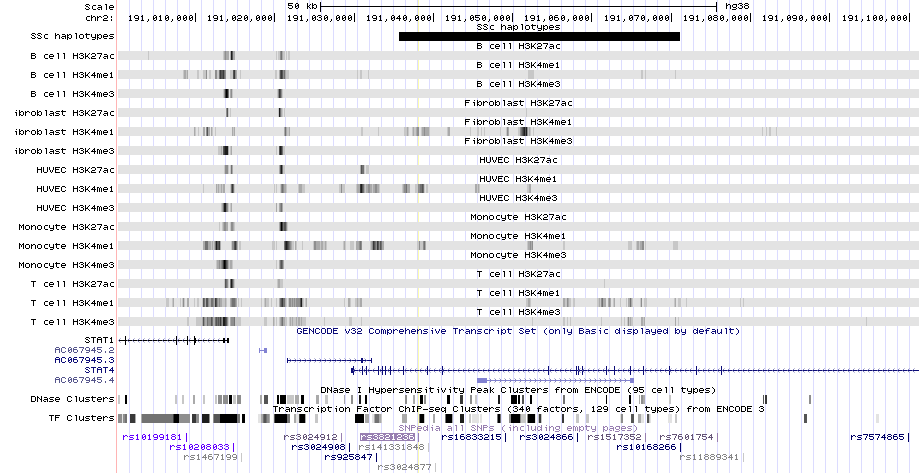
**

1. **SCHIP1-IL12A**

**
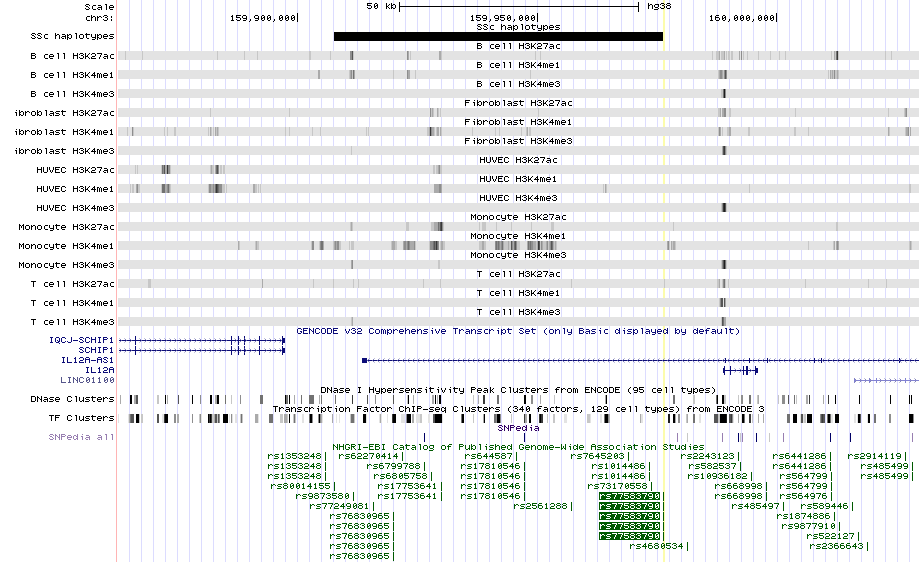
**

1. **ATG5**

**
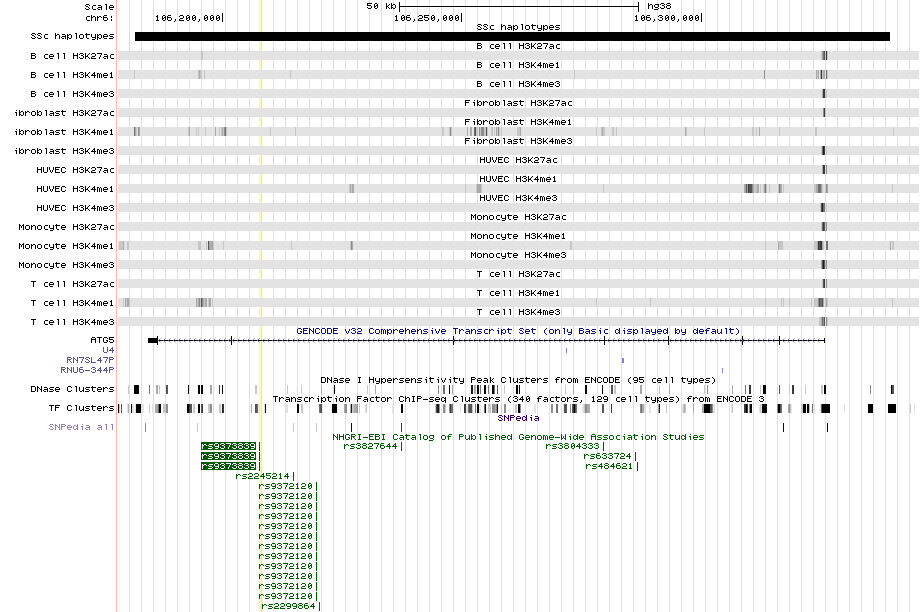
**

1. **IRF5**

**
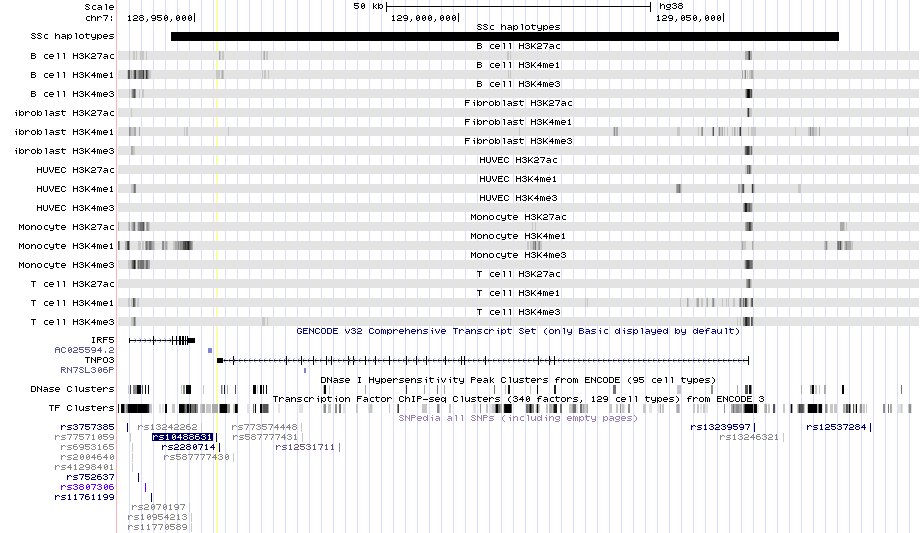
**

1. **CSK**

**
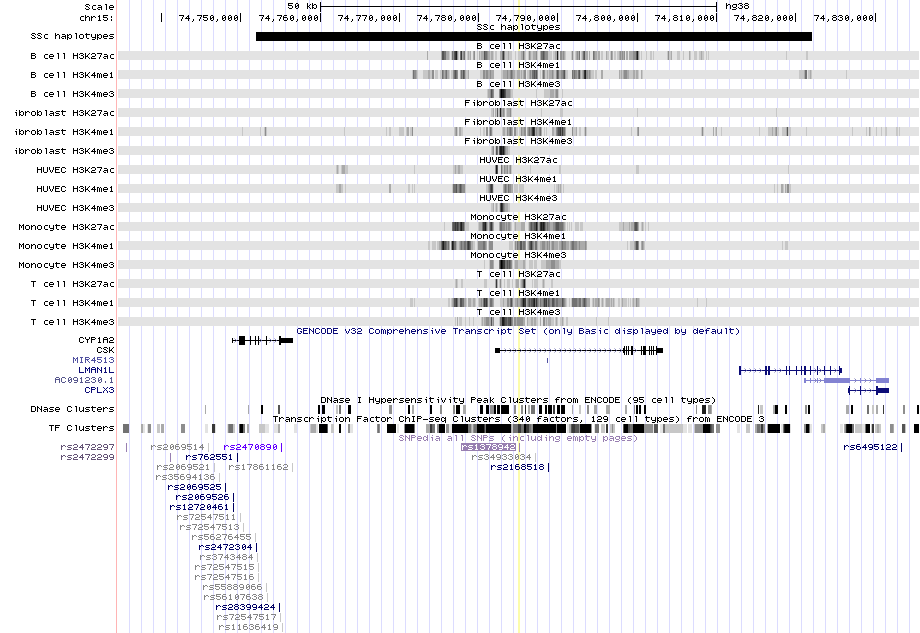
**

1. **IL12RB1**

**
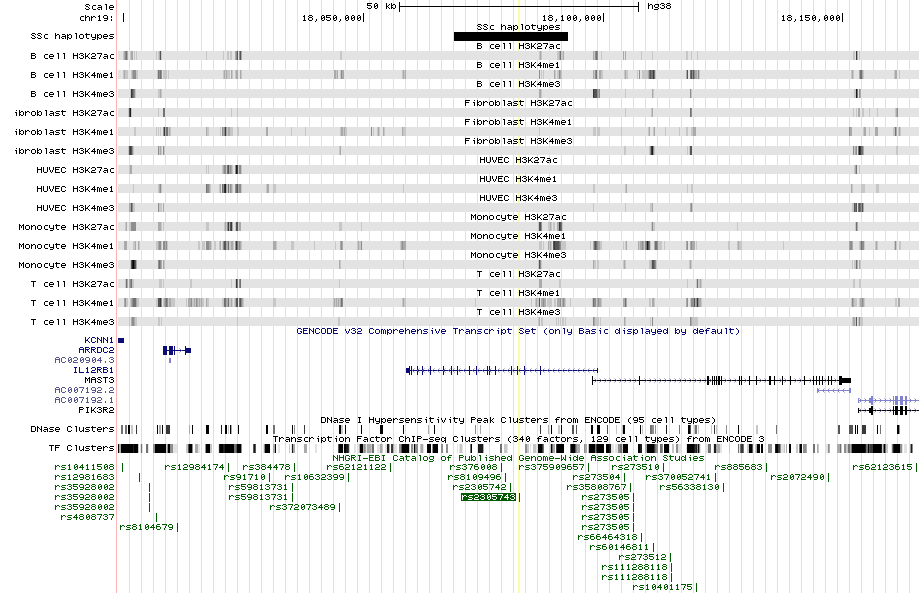
**
