# Supplementary material for: Broadening our understanding of genetic risk for scleroderma/systemic sclerosis by querying the chromatin architecture surrounding the risk haplotypes
Source: BMC Med Genomics. 2021 Apr 24;14:114. doi: 10.1186/s12920-021-00964-5 (PMC8066847; doi:10.1186/s12920-021-00964-5)
Supplement: Supplementary file 2 — Additional file 2. Significant biological process, molecular function, and cellular component GORILLA ontologies. [file 12920_2021_964_MOESM2_ESM.docx]

**Additional File 2: Significant biological process, molecular function, and cellular component GORILLA ontologies associated with genes expressed within SSc-risk TADs.**

| **CD4+ T Cells**  **Biological Process** | | |
| --- | --- | --- |
| **GO Term** | **Description** | **q-value** |
| GO:0051252 | regulation of RNA metabolic process | 0.0000127 |
| GO:0006357 | regulation of transcription by RNA polymerase II | 0.0000133 |
| GO:0006355 | regulation of transcription, DNA-templated | 0.0000188 |
| GO:1903506 | regulation of nucleic acid-templated transcription | 0.0000144 |
| GO:2001141 | regulation of RNA biosynthetic process | 0.0000138 |
| GO:0019219 | regulation of nucleobase-containing compound metabolic process | 0.0000215 |
| GO:0010468 | regulation of gene expression | 0.0000225 |
| GO:0010556 | regulation of macromolecule biosynthetic process | 0.0000416 |
| GO:2000112 | regulation of cellular macromolecule biosynthetic process | 0.000037 |
| GO:0031326 | regulation of cellular biosynthetic process | 0.000177 |
| GO:0009889 | regulation of biosynthetic process | 0.000324 |
| GO:0060255 | regulation of macromolecule metabolic process | 0.000613 |
| GO:0051171 | regulation of nitrogen compound metabolic process | 0.00296 |
| GO:0010629 | negative regulation of gene expression | 0.00365 |
| GO:2000113 | negative regulation of cellular macromolecule biosynthetic process | 0.00681 |
| GO:0045892 | negative regulation of transcription, DNA-templated | 0.00683 |
| GO:1903507 | negative regulation of nucleic acid-templated transcription | 0.00677 |
| GO:1902679 | negative regulation of RNA biosynthetic process | 0.00672 |
| GO:0010558 | negative regulation of macromolecule biosynthetic process | 0.00698 |
| GO:0051253 | negative regulation of RNA metabolic process | 0.00758 |
| GO:0019222 | regulation of metabolic process | 0.00803 |
| GO:0080090 | regulation of primary metabolic process | 0.00775 |
| GO:0031327 | negative regulation of cellular biosynthetic process | 0.00745 |
| GO:0009890 | negative regulation of biosynthetic process | 0.0124 |
| GO:0031323 | regulation of cellular metabolic process | 0.0225 |
| GO:0000122 | negative regulation of transcription by RNA polymerase II | 0.0278 |
| GO:0090304 | nucleic acid metabolic process | 0.04 |
| GO:0045934 | negative regulation of nucleobase-containing compound metabolic process | 0.0498 |
| GO:0016070 | RNA metabolic process | 0.0888 |
| GO:0010605 | negative regulation of macromolecule metabolic process | 0.115 |
| GO:0015031 | protein transport | 0.112 |
| GO:0015833 | peptide transport | 0.182 |
| GO:0042886 | amide transport | 0.191 |
| GO:0006139 | nucleobase-containing compound metabolic process | 0.27 |
| GO:0045184 | establishment of protein localization | 0.282 |
| GO:0032436 | positive regulation of proteasomal ubiquitin-dependent protein catabolic process | 0.28 |
| GO:0009892 | negative regulation of metabolic process | 0.285 |
| GO:0002731 | negative regulation of dendritic cell cytokine production | 0.315 |
| GO:0008612 | peptidyl-lysine modification to peptidyl-hypusine | 0.306 |
| GO:1903364 | positive regulation of cellular protein catabolic process | 0.356 |
| GO:1901800 | positive regulation of proteasomal protein catabolic process | 0.356 |
| **Molecular Function** | | |
| **GO Term** | **Description** | **q-value** |
| GO:0003677 | DNA binding | 5.55E-08 |
| GO:0003676 | nucleic acid binding | 7.33E-08 |
| GO:0140110 | transcription regulator activity | 7.96E-08 |
| GO:1901363 | heterocyclic compound binding | 0.00000201 |
| GO:0044212 | transcription regulatory region DNA binding | 0.00000527 |
| GO:0001067 | regulatory region nucleic acid binding | 0.00000452 |
| GO:0097159 | organic cyclic compound binding | 0.00000442 |
| GO:0043565 | sequence-specific DNA binding | 0.0000075 |
| GO:0000977 | RNA polymerase II regulatory region sequence-specific DNA binding | 0.00000875 |
| GO:0001012 | RNA polymerase II regulatory region DNA binding | 0.00000788 |
| GO:0000976 | transcription regulatory region sequence-specific DNA binding | 0.0000105 |
| GO:0003700 | DNA-binding transcription factor activity | 0.0000267 |
| GO:1990837 | sequence-specific double-stranded DNA binding | 0.0000575 |
| GO:0000981 | DNA-binding transcription factor activity, RNA polymerase II-specific | 0.0000535 |
| GO:0003690 | double-stranded DNA binding | 0.0000889 |
| GO:0005488 | binding | 0.000144 |
| GO:0005515 | protein binding | 0.000385 |
| GO:0001227 | DNA-binding transcription repressor activity, RNA polymerase II-specific | 0.013 |
| GO:0003674 | molecular_function | 0.0769 |
| GO:0045125 | bioactive lipid receptor activity | 0.149 |
| GO:0031489 | myosin V binding | 0.185 |
| **Cellular Component** | |  |
| **GO** | **Description** | **q-value** |
| GO:0044424 | intracellular part | 1.53E-08 |
| GO:0043231 | intracellular membrane-bounded organelle | 3.13E-08 |
| GO:0005634 | nucleus | 2.75E-08 |
| GO:0043227 | membrane-bounded organelle | 2.89E-08 |
| GO:0043229 | intracellular organelle | 2.51E-08 |
| GO:0043226 | organelle | 2.67E-07 |
| GO:0044428 | nuclear part | 0.000426 |
| GO:0005654 | nucleoplasm | 0.00131 |
| GO:1990904 | ribonucleoprotein complex | 0.0843 |
| GO:0044464 | cell part | 0.108 |
| GO:0001891 | phagocytic cup | 0.119 |
| GO:0042022 | interleukin-12 receptor complex | 0.126 |

| **Monocytes**  **Biological Process** | | |
| --- | --- | --- |
| **GO Term** | **Description** | **q-value** |
| GO:0001817 | regulation of cytokine production | 1.02E-02 |
| GO:0070757 | interleukin-35-mediated signaling pathway | 3.16E-02 |
| GO:0001819 | positive regulation of cytokine production | 2.27E-02 |
| GO:0032729 | positive regulation of interferon-gamma production | 1.63E-01 |
| GO:0060333 | interferon-gamma-mediated signaling pathway | 1.85E-01 |
| GO:1901652 | response to peptide | 2.37E-01 |
| GO:0019221 | cytokine-mediated signaling pathway | 2.93E-01 |
| GO:0050691 | regulation of defense response to virus by host | 3.82E-01 |
| GO:0032735 | positive regulation of interleukin-12 production | 3.96E-01 |
| GO:0038114 | interleukin-21-mediated signaling pathway | 3.74E-01 |
| GO:0032649 | regulation of interferon-gamma production | 3.69E-01 |
| GO:0043382 | positive regulation of memory T cell differentiation | 4.00E-01 |
| GO:0038155 | interleukin-23-mediated signaling pathway | 3.70E-01 |
| GO:0035722 | interleukin-12-mediated signaling pathway | 4.12E-01 |
| GO:0032479 | regulation of type I interferon production | 4.97E-01 |
| GO:0043380 | regulation of memory T cell differentiation | 5.47E-01 |
| GO:0033962 | cytoplasmic mRNA processing body assembly | 6.08E-01 |
| GO:0032655 | regulation of interleukin-12 production | 6.26E-01 |
| GO:0045806 | negative regulation of endocytosis | 6.85E-01 |
| GO:0001817 | regulation of cytokine production | 1.02E-02 |
| GO:0070757 | interleukin-35-mediated signaling pathway | 3.16E-02 |
| **Molecular Function** | | |
| **GO Term** | **Description** | **q-value** |
| [GO:0000979](http://www.godatabase.org/cgi-bin/amigo/go.cgi?query=GO:0000979&view=details) | RNA polymerase II core promoter sequence-specific DNA binding | 1.00E+00 |
| **Cellular Component** | |  |
| **GO Term** | **Description** | **q-value** |
| [GO:0005751](http://www.godatabase.org/cgi-bin/amigo/go.cgi?query=GO:0005751&view=details) | mitochondrial respiratory chain complex IV | 9.25E-01 |
