# Supplementary material for: Broadening our understanding of genetic risk for scleroderma/systemic sclerosis by querying the chromatin architecture surrounding the risk haplotypes
Source: BMC Med Genomics. 2021 Apr 24;14:114. doi: 10.1186/s12920-021-00964-5 (PMC8066847; doi:10.1186/s12920-021-00964-5)
Supplement: Supplementary file 3 — Additional file 3. Significant disease and biological function IPA annotations. [file 12920_2021_964_MOESM3_ESM.docx]

**Additional File 3: Significant disease and biological function IPA annotations associated with genes expressed within SSc-risk TADs.**

**CD4+ T Cells**

| **Categories** | **Diseases or Functions Annotation** | **q-value** |
| --- | --- | --- |
| Cancer,Organismal Injury and Abnormalities | Nonhematologic malignant neoplasm | 4.54E-27 |
| Cancer,Organismal Injury and Abnormalities | Cancer | 1.34E-25 |
| Cancer,Organismal Injury and Abnormalities | Malignant solid tumor | 1.34E-25 |
| Cancer,Organismal Injury and Abnormalities | Non-melanoma solid tumor | 2.5E-25 |
| Cancer,Organismal Injury and Abnormalities | Head and neck tumor | 2.5E-25 |
| Cancer,Organismal Injury and Abnormalities | Carcinoma | 8.51E-25 |
| Cancer,Organismal Injury and Abnormalities | Head and neck cancer | 8.93E-24 |
| Cancer,Organismal Injury and Abnormalities | Head and neck carcinoma | 4.67E-23 |
| Cancer,Organismal Injury and Abnormalities | Extracranial solid tumor | 8.52E-23 |
| Cancer,Organismal Injury and Abnormalities | Neck neoplasm | 2.08E-22 |
| Cancer,Endocrine System Disorders,Organismal Injury and Abnormalities | Thyroid carcinoma | 2.43E-22 |
| Cancer,Endocrine System Disorders,Organismal Injury and Abnormalities | Endocrine carcinoma | 7.79E-20 |
| Cancer,Organismal Injury and Abnormalities | Cancer of secretory structure | 2.78E-19 |
| Cancer,Organismal Injury and Abnormalities | Formation of solid tumor | 4.53E-17 |
| Cancer,Organismal Injury and Abnormalities | Abdominal carcinoma | 7.99E-16 |
| Cancer,Gastrointestinal Disease,Organismal Injury and Abnormalities | Large intestine carcinoma | 7.99E-16 |
| Cancer,Gastrointestinal Disease,Organismal Injury and Abnormalities | Large intestine adenocarcinoma | 7.99E-16 |
| Cancer,Gastrointestinal Disease,Organismal Injury and Abnormalities | Gastrointestinal adenocarcinoma | 9.59E-16 |
| Cancer,Gastrointestinal Disease,Organismal Injury and Abnormalities | Gastrointestinal carcinoma | 1.99E-15 |
| Cancer,Gastrointestinal Disease,Organismal Injury and Abnormalities | Gastrointestinal tumor | 1.51E-14 |
| Cancer,Organismal Injury and Abnormalities | Abdominal cancer | 2.34E-14 |
| Cancer,Organismal Injury and Abnormalities | Abdominal neoplasm | 2.65E-14 |
| Cancer,Gastrointestinal Disease,Organismal Injury and Abnormalities | Gastrointestinal tract cancer | 3.15E-14 |
| Cancer,Neurological Disease,Organismal Injury and Abnormalities | Central nervous system cancer | 1.33E-13 |
| Cancer,Neurological Disease,Organismal Injury and Abnormalities | Brain glioma | 1.34E-13 |
| Neurological Disease,Organismal Injury and Abnormalities | Brain lesion | 1.39E-13 |
| Cancer,Organismal Injury and Abnormalities | Abdominal adenocarcinoma | 1.51E-13 |
| Cancer,Neurological Disease,Organismal Injury and Abnormalities | Glioma cancer | 1.51E-13 |
| Cancer,Neurological Disease,Organismal Injury and Abnormalities | Brain tumor | 1.56E-13 |
| Cancer,Organismal Injury and Abnormalities | Intraabdominal organ tumor | 2E-13 |
| Cancer,Neurological Disease,Organismal Injury and Abnormalities | Central nervous system solid tumor | 2.18E-13 |
| Cancer,Organismal Injury and Abnormalities | Adenocarcinoma | 2.94E-13 |
| Cancer,Neurological Disease,Organismal Injury and Abnormalities | Nervous system neoplasm | 3.61E-13 |
| Cancer,Gastrointestinal Disease,Organismal Injury and Abnormalities | Malignant neoplasm of large intestine | 3.73E-13 |
| Cancer,Gastrointestinal Disease,Organismal Injury and Abnormalities | Digestive organ tumor | 4.01E-13 |
| Cancer,Gastrointestinal Disease,Organismal Injury and Abnormalities | Intestinal tumor | 4.8E-13 |
| Cancer,Neurological Disease,Organismal Injury and Abnormalities | Glioma | 7.05E-13 |
| Cancer,Gastrointestinal Disease,Organismal Injury and Abnormalities | Digestive system cancer | 7.12E-12 |
| Cancer,Neurological Disease,Organismal Injury and Abnormalities | Grade 3-4 glioma | 5.61E-07 |
| Cancer,Neurological Disease,Organismal Injury and Abnormalities | Grade 3-4 glioma cancer | 8.73E-07 |
| Cancer,Organismal Injury and Abnormalities | Incidence of tumor | 0.00000317 |
| Cancer,Organismal Injury and Abnormalities | Development of malignant tumor | 0.00000491 |
| Cancer,Organismal Injury and Abnormalities | Frequency of tumor | 0.00000519 |
| Cancer,Organismal Injury and Abnormalities | Development of carcinoma | 0.0000104 |
| Cancer,Organismal Injury and Abnormalities | Tumorigenesis of epithelial neoplasm | 0.0000149 |
| Cancer,Neurological Disease,Organismal Injury and Abnormalities | Grade 1-4 astrocytoma | 0.0000161 |
| Cancer,Neurological Disease,Organismal Injury and Abnormalities | Grade 4 high grade glioma | 0.0000161 |
| Cancer,Neurological Disease,Organismal Injury and Abnormalities | Grade 4 malignant glioma | 0.0000161 |
| Cancer,Neurological Disease,Organismal Injury and Abnormalities | Grade 4 astrocytoma | 0.0000161 |
| Cancer,Neurological Disease,Organismal Injury and Abnormalities | Brain astrocytoma | 0.0000186 |
| Cancer,Neurological Disease,Organismal Injury and Abnormalities | High grade astrocytoma | 0.0000215 |
| Cancer,Organismal Injury and Abnormalities | Cancer of head | 0.0000275 |
| Cancer,Neurological Disease,Organismal Injury and Abnormalities | Gliomatosis cerebri | 0.0000448 |
| Cancer,Neurological Disease,Organismal Injury and Abnormalities | Brain cancer | 0.0000781 |
| Cancer,Organismal Injury and Abnormalities | Malignant solid organ tumor | 0.000185 |
| Cell-mediated Immune Response,Cellular Development,Cellular Function and Maintenance,Cellular Growth and Proliferation,Embryonic Development,Hematological System Development and Function,Hematopoiesis,Lymphoid Tissue Structure and Development,Organ Development,Organismal Development,Tissue Development | Differentiation of T lymphocytes | 0.000187 |
| Infectious Diseases | Replication of RNA virus | 0.000187 |
| Infectious Diseases | Replication of virus | 0.000372 |
| Gene Expression | Transcription of RNA | 0.000372 |
| Neurological Disease,Organismal Injury and Abnormalities | Cerebral disorder | 0.000385 |
| Cancer,Dermatological Diseases and Conditions,Organismal Injury and Abnormalities | Skin tumor | 0.000629 |
| Protein Synthesis | Metabolism of protein | 0.000644 |
| Cancer,Dermatological Diseases and Conditions,Organismal Injury and Abnormalities | Skin cancer | 0.000654 |
| Cell-mediated Immune Response,Cellular Function and Maintenance,Hematological System Development and Function | T cell homeostasis | 0.000797 |
| Cellular Function and Maintenance | Lymphocyte homeostasis | 0.000977 |
| Cancer,Organismal Injury and Abnormalities | Genitourinary tumor | 0.00102 |
| Cellular Development,Cellular Growth and Proliferation,Hematological System Development and Function,Lymphoid Tissue Structure and Development | Proliferation of lymphocytes | 0.00108 |
| Cellular Development,Cellular Growth and Proliferation,Hematological System Development and Function,Lymphoid Tissue Structure and Development | Cell proliferation of T lymphocytes | 0.00108 |
| Cell-mediated Immune Response,Cellular Development,Cellular Function and Maintenance,Cellular Growth and Proliferation,Embryonic Development,Hematological System Development and Function,Hematopoiesis,Lymphoid Tissue Structure and Development,Organ Development,Organismal Development,Tissue Development | T cell development | 0.0011 |
| Infectious Diseases | Replication of Influenza A virus | 0.00128 |
| Cancer,Organismal Injury and Abnormalities | Pelvic tumor | 0.00143 |
| Cancer,Dermatological Diseases and Conditions,Organismal Injury and Abnormalities | Cutaneous melanoma | 0.0015 |
| Cellular Development,Cellular Growth and Proliferation,Hematological System Development and Function,Lymphoid Tissue Structure and Development | Proliferation of immune cells | 0.00157 |
| Cancer,Organismal Injury and Abnormalities | Melanoma | 0.00183 |
| Cancer,Organismal Injury and Abnormalities | Malignant genitourinary solid tumor | 0.002 |
| Cancer,Organismal Injury and Abnormalities | Pelvic carcinoma | 0.00215 |
| Cancer,Organismal Injury and Abnormalities | Genitourinary carcinoma | 0.0022 |
| Cancer,Gastrointestinal Disease,Organismal Injury and Abnormalities | Development of digestive organ tumor | 0.0024 |
| Cell-mediated Immune Response,Cellular Development,Cellular Function and Maintenance,Cellular Growth and Proliferation,Embryonic Development,Hematological System Development and Function,Hematopoiesis,Lymphoid Tissue Structure and Development,Organ Development,Organismal Development,Tissue Development | Differentiation of helper T lymphocytes | 0.00251 |
| Cellular Development,Cellular Growth and Proliferation,Embryonic Development,Hematological System Development and Function,Hematopoiesis,Lymphoid Tissue Structure and Development,Organ Development,Organismal Development,Tissue Development | Lymphopoiesis | 0.00292 |
| Cellular Development,Cellular Growth and Proliferation | Proliferation of blood cells | 0.00299 |
| Cellular Development,Cellular Growth and Proliferation,Hematological System Development and Function,Hematopoiesis,Lymphoid Tissue Structure and Development,Tissue Development | Leukopoiesis | 0.00354 |
| Gene Expression | Transcription of DNA | 0.00379 |
| Cancer,Organismal Injury and Abnormalities | Pelvic cancer | 0.0039 |
| RNA Post-Transcriptional Modification | Processing of RNA | 0.00455 |
| Gene Expression | Expression of RNA | 0.00514 |
| Cell-To-Cell Signaling and Interaction,Hematological System Development and Function,Immune Cell Trafficking,Inflammatory Response | Activation of T lymphocytes | 0.00557 |
| Cell Cycle,Gene Expression | Binding of DNA | 0.00625 |
| Cancer,Gastrointestinal Disease,Organismal Injury and Abnormalities | Tumorigenesis of gastrointestinal tumor | 0.00748 |
| Cancer,Gastrointestinal Disease,Organismal Injury and Abnormalities | Colon carcinoma | 0.00864 |
| Cancer,Organismal Injury and Abnormalities,Reproductive System Disease | Genital tumor | 0.00864 |
| Cell Death and Survival | Cell viability | 0.00949 |

**Monocytes**

| **Categories** | **Diseases or Functions Annotation** | **q-value** |
| --- | --- | --- |
| Cell-To-Cell Signaling and Interaction,Hematological System Development and Function,Immune Cell Trafficking,Inflammatory Response | Activation of lymphocytes | 1.38E-05 |
| Cell-mediated Immune Response,Cellular Development,Cellular Function and Maintenance,Cellular Growth and Proliferation,Embryonic Development,Hematological System Development and Function,Hematopoiesis,Lymphoid Tissue Structure and Development,Organ Development,Organismal Development,Tissue Development | T cell development | 4.32E-05 |
| Connective Tissue Disorders,Immunological Disease,Inflammatory Disease,Organismal Injury and Abnormalities,Skeletal and Muscular Disorders | Systemic lupus erythematosus | 4.32E-05 |
| Cell-mediated Immune Response,Cellular Development,Cellular Function and Maintenance,Cellular Growth and Proliferation,Embryonic Development,Hematological System Development and Function,Hematopoiesis,Lymphoid Tissue Structure and Development,Organ Development,Organismal Development,Tissue Development | Differentiation of T lymphocytes | 5.50E-05 |
| Cell-mediated Immune Response,Cellular Development,Cellular Function and Maintenance,Cellular Growth and Proliferation,Embryonic Development,Hematological System Development and Function,Hematopoiesis,Lymphoid Tissue Structure and Development,Organ Development,Organismal Development,Tissue Development | Differentiation of Th1 cells | 9.44E-05 |
| Cell-To-Cell Signaling and Interaction,Hematological System Development and Function,Immune Cell Trafficking,Inflammatory Response | Activation of T lymphocytes | 9.44E-05 |
| Cell-mediated Immune Response,Cellular Development,Cellular Function and Maintenance,Cellular Growth and Proliferation,Embryonic Development,Hematological System Development and Function,Hematopoiesis,Lymphoid Tissue Structure and Development,Organ Development,Organismal Development,Tissue Development | Differentiation of helper T lymphocytes | 9.44E-05 |
| Cellular Development,Cellular Growth and Proliferation,Hematological System Development and Function,Lymphoid Tissue Structure and Development | Cell proliferation of T lymphocytes | 3.71E-04 |
| Cell-mediated Immune Response,Cellular Development,Cellular Function and Maintenance,Cellular Growth and Proliferation,Embryonic Development,Hematological System Development and Function,Hematopoiesis,Lymphoid Tissue Structure and Development,Organ Development,Organismal Development,Tissue Development | Development of helper T lymphocytes | 5.96E-04 |
| Gastrointestinal Disease,Inflammatory Disease | Inflammatory Bowel Disease | 7.28E-04 |
| Cancer,Hematological Disease,Organismal Injury and Abnormalities | Myeloproliferative neoplasm | 7.28E-04 |
| Cellular Function and Maintenance | Function of lymphatic system cells | 1.02E-03 |
| Cancer,Hematological Disease,Organismal Injury and Abnormalities | Chronic myeloproliferative neoplasm | 1.30E-03 |
| Hematological System Development and Function,Tissue Morphology | Quantity of blood cells | 1.30E-03 |
| Hematological System Development and Function,Lymphoid Tissue Structure and Development,Organ Development,Tissue Development | Growth of lymphoid organ | 1.67E-03 |
| Hematological Disease,Infectious Diseases | Endotoxemia | 1.76E-03 |
| Infectious Diseases | Sepsis | 2.10E-03 |
| Cellular Function and Maintenance | Function of leukocytes | 2.11E-03 |
| Cell-To-Cell Signaling and Interaction | Activation of cells | 2.11E-03 |
| Hematological System Development and Function,Tissue Morphology | Quantity of mononuclear leukocytes | 2.21E-03 |
| Lymphoid Tissue Structure and Development,Tissue Morphology | Quantity of lymphatic system cells | 2.82E-03 |
| Gastrointestinal Disease,Inflammatory Disease | Crohn disease | 2.92E-03 |
| Cellular Function and Maintenance,Hematological System Development and Function | Function of lymphocytes | 2.92E-03 |
| Hematological System Development and Function,Tissue Morphology | Quantity of leukocytes | 3.35E-03 |
| Cell Death and Survival | Cell death of lymphoma cell lines | 3.74E-03 |
| Cell Death and Survival,Cellular Compromise | Cytotoxicity of cells | 3.98E-03 |
| Hematological System Development and Function,Tissue Morphology | Quantity of antigen presenting cells | 4.41E-03 |
| Hematological System Development and Function,Lymphoid Tissue Structure and Development,Tissue Morphology | Quantity of lymphocytes | 4.53E-03 |
| Cancer,Hematological Disease,Immunological Disease,Organismal Injury and Abnormalities | T-cell non-Hodgkin lymphoma | 4.59E-03 |
| Hematological System Development and Function,Inflammatory Response,Tissue Morphology | Quantity of macrophages | 5.13E-03 |
| Cell Death and Survival | Cell death of immune cells | 5.44E-03 |
| Cancer,Hematological Disease,Organismal Injury and Abnormalities | Mature T-cell or NK-cell neoplasm | 5.58E-03 |
| Cell Death and Survival,Cellular Compromise | Cytotoxicity of lymphatic system cells | 6.30E-03 |
| Cancer,Organismal Injury and Abnormalities | Adult solid tumor | 6.30E-03 |
| Hematological System Development and Function,Hematopoiesis,Tissue Morphology | Quantity of hematopoietic progenitor cells | 6.55E-03 |
| Organismal Survival | Survival of organism | 7.09E-03 |
| Cancer,Hematological Disease,Immunological Disease,Organismal Injury and Abnormalities | Natural killer cell lymphoma | 7.21E-03 |
| Hematological System Development and Function,Inflammatory Response,Tissue Morphology | Quantity of phagocytes | 7.37E-03 |
| Hematological System Development and Function,Tissue Morphology | Quantity of myeloid cells | 7.37E-03 |
| Cancer,Hematological Disease,Immunological Disease,Organismal Injury and Abnormalities | Chronic myeloid leukemia | 7.83E-03 |
| Cell Death and Survival | Cytolysis | 8.47E-03 |
| Cellular Development,Cellular Growth and Proliferation,Hematological System Development and Function,Hematopoiesis | Proliferation of hematopoietic progenitor cells | 8.86E-03 |
| Cancer,Organismal Injury and Abnormalities | Carcinoma | 9.33E-03 |
