# Supplementary material for: Broadening our understanding of genetic risk for scleroderma/systemic sclerosis by querying the chromatin architecture surrounding the risk haplotypes
Source: BMC Med Genomics. 2021 Apr 24;14:114. doi: 10.1186/s12920-021-00964-5 (PMC8066847; doi:10.1186/s12920-021-00964-5)
Supplement: Supplementary file 4 — Additional file 4. Histone marks present in expanded scleroderma-associated haplotypes. [file 12920_2021_964_MOESM4_ESM.docx]

**Additional File 4. Histone marks present in expanded scleroderma-associated haplotypes.**

| **Haplotype Block** | **SNP** | **B cell** | | | **Fibroblast** | | | **HUVEC** | | | **Monocyte** | | | **T cell** | | |
| --- | --- | --- | --- | --- | --- | --- | --- | --- | --- | --- | --- | --- | --- | --- | --- | --- |
|  |  | **H3K27ac** | **H3K4me1** | **H3K4me3** | **H3K27ac** | **H3K4me1** | **H3K4me3** | **H3K27ac** | **H3K4me1** | **H3K4me3** | **H3K27ac** | **H3K4me1** | **H3K4me3** | **H3K27ac** | **H3K4me1** | **H3K4me3** |
|  | **p-value** | **0.148** | **0.081** | **0.060** | **0.113** | **0.192** | **0.413** | **0.072** | **0.031** | **0.037** | **0.019** | **0.080** | **0.075** | **0.326** | **0.309** | **0.221** |
| chr1:114846430-114920190 | rs2476601 | – | Y | – | Y | Y | – | – | Y | Y | – | Y | Y | – | Y | Y |
| chr1:173160614-173225689 | rs2205960 | Y | Y | Y | Y | Y | – | Y | Y | Y | Y | Y | Y | Y | Y | Y |
| chr1:36179795-36571854 | rs2275247 | Y | Y | Y | Y | Y | Y | Y | Y | Y | Y | Y | Y | Y | Y | Y |
| chr1:68271489-68292699 | rs3790567 | – | – | – | – | – | – | – | – | – | Y | Y | Y | – | – | – |
| chr1:167387121-167405537 | rs2056626 | – | – | – | – | – | – | Y | Y | – | Y | – | – | – | Y | – |
| chr1:68161062-68209235 | rs11465804 | – | – | – | Y | Y | – | Y | Y | Y | – | – | – | – | – | – |
| chr2:192808468-192837760 | rs11889341 | – | – | – | – | – | – | – | – | – | – | – | – | – | – | – |
| chr3:58357059-58448359 | rs4681851 | Y | Y | Y | Y | Y | Y | Y | Y | Y | Y | Y | Y | Y | Y | Y |
| chr3:159343182-159411842 | rs77583790 | – | – | – | Y | Y | – | – | – | – | – | – | – | – | – | – |
| chr3:12239497-12315173 | rs310746 | – | Y | – | Y | Y | Y | – | Y | Y | Y | Y | Y | – | Y | Y |
| chr4:103657900-103679560 | rs17266594 | – | – | – | – | – | – | – | – | – | – | – | – | – | – | – |
| chr4:123948878-124422241 | rs2069762 | Y | Y | Y | Y | Y | – | Y | Y | Y | Y | Y | Y | – | – | – |
| chr4:124427211-124472187 | rs907715 | – | – | – | Y | Y | – | Y | Y | Y | – | – | – | – | – | – |
| chr4:104320206-104455717 | rs1598859 | – | – | – | – | – | – | – | – | – | – | – | – | – | – | – |
| chr4:155536047-155615731 | rs5743704 | – | – | – | Y | Y | – | Y | Y | Y | – | Y | Y | – | – | – |
| chr5:149812422-149830638 | rs2233287 | – | – | – | – | – | – | Y | Y | – | – | Y | – | – | – | – |
| chr6:107077565-107235044 | rs9373839 | – | Y | Y | Y | Y | Y | – | Y | Y | Y | Y | Y | Y | Y | Y |
| chr6:138492603-138563574 | rs5029939 | – | Y | Y | Y | Y | – | Y | Y | Y | Y | Y | Y | – | – | – |
| chr7:128225670-128351928 | rs10488631 | Y | Y | Y | Y | Y | Y | Y | Y | Y | Y | Y | Y | Y | Y | Y |
| chr7:28212351-28239716 | rs1635852 | – | – | – | – | – | – | – | Y | – | Y | Y | Y | – | – | – |
| chr8:11195096-11210509 | rs2736340 | Y | Y | Y | Y | Y | Y | Y | Y | Y | Y | Y | Y | Y | Y | – |
| chr8:18450283-18456107 | rs10096702 | – | – | – | – | – | – | – | – | – | – | – | – | – | – | – |
| chr10:6120516-6139246 | rs12722495 | – | – | – | Y | Y | – | Y | Y | – | Y | Y | Y | – | Y | Y |
| chr11:575408-634083 | rs1131665 | Y | Y | Y | Y | – | Y | Y | Y | Y | Y | Y | Y | Y | Y | Y |
| chr11:102590265-102648034 | rs2276109 | – | Y | – | Y | Y | – | Y | Y | Y | – | – | – | – | – | – |
| chr14:105852376-105874519 | rs2841277 | Y | Y | Y | – | – | – | – | – | – | – | – | – | – | – | – |
| chr15:75336579-75406663 | rs1378942 | Y | Y | Y | Y | Y | Y | Y | Y | Y | Y | Y | Y | Y | Y | Y |
| chr16:86051321-86052857 | rs2280381 | – | – | – | Y | Y | – | – | – | – | – | – | – | – | – | – |
| chr16:31281493-31357099 | rs1143679 | – | Y | Y | Y | – | – | – | Y | – | Y | Y | Y | Y | Y | Y |
| chr18:65178882-65210925 | rs727088 | – | – | – | – | – | – | – | – | – | – | – | – | – | – | – |
| chr19:18290482-18313779 | rs2305743 | Y | Y | Y | Y | Y | – | Y | Y | Y | Y | Y | Y | Y | Y | Y |
| chr19:44675295-44683913 | rs344781 | – | – | – | Y | Y | – | Y | Y | – | Y | Y | – | – | – | – |
| chr22:24630461-24634047 | rs755622 | – | – | – | Y | – | – | – | – | – | – | – | – | – | – | – |
